# Role of microencapsulated *Lactobacillus plantarum* in alleviating intestinal inflammatory damage through promoting epithelial proliferation and differentiation in layer chicks

**DOI:** 10.3389/fmicb.2023.1287899

**Published:** 2023-11-20

**Authors:** Yaoming Cui, Peiyu Huang, Haitao Duan, Shijia Song, Liping Gan, Zhen Liu, Qiaohan Lin, Jinrong Wang, Gunghai Qi, Junjun Guan

**Affiliations:** ^1^School of Biological Engineering, Henan University of Technology, Zhengzhou, Henan, China; ^2^College of Animal Science and Technology, Henan University of Animal Husbandry and Economy, Zhengzhou, Henan, China; ^3^Feed Research Institute, Chinese Academy of Agricultural Sciences, Beijing, China

**Keywords:** intestinal development, proliferation and differentiation, microbiota, layer chick, *Lactobacillus plantarum*

## Abstract

The alleviating effects of *Lactobacillus plantarum* in microencapsulation (LPM) on lipopolysaccharide (LPS)-induced intestinal inflammatory injury were investigated in layer chicks. A total of 252 healthy Hy-Line Brown layer chicks were randomly divided into six groups. Birds were injected with saline or LPS except for the control, and the diets of birds subjected to LPS were supplemented with nothing, *L. plantarum*, LPM, and wall material of LPM, respectively. The viable counts of LPM reached 10^9^ CFU/g, and the supplemental levels of *L. plantarum*, LPM, and WM were 0.02 g (10^9^ CFU), 1.0 g, and 0.98 g, per kilogram feed, respectively. LPS administration caused intestinal damage in layer chicks, evidenced by increased proinflammatory factors accompanied by poor intestinal development and morphology (*p* < 0.05). LPM/LPS significantly increased body weight, small intestine weight and length, villus height, villus height/crypt depth, and mRNA relative expression of tight junction protein genes (*p* < 0.05) and performed better than free *L. plantarum*. These findings could be attributed to the significant increase in viable counts of *L. plantarum* in the small intestine (*p* < 0.05), as well as the enhanced levels of Actinobacteriota, Lactobacillaceae, and *Lactobacillus* in intestinal microbiota (*p* < 0.05). Such results could further significantly increase goblet and PCNA+ cell percentage (*p* < 0.05); the mRNA relative expressions of epithelial cell, fast-cycling stem cell, quiescent stem cell, endocrine cell, and Paneth cell; and goblet and proliferative cell marker genes, including *E-cadherin*, *Lgr-5*, *Bmi-1*, *ChA*, *Lysozome*, *Mucin-2*, and *PCNA* (*p* < 0.05). Furthermore, the mRNA relative expressions of key genes involved in epithelial cell proliferation, namely, *c-Myc*, *Cyclin-1*, *Wnt-3*, *Lrp-5,* and *Olfm-4*, exhibited significant upregulation compared with the LPS treatment, as well as the differentiating genes *Notch-1* and *Hes-1* (*p* < 0.05). To sum up, microencapsulated *L. plantarum* supplementation could alleviate intestinal injury in layer chicks induced by LPS by promoting the proliferation and differentiation of intestinal epithelial cells, which could be attributed to the increase in viable count of *L. plantarum* in the gut and optimization in intestinal microbial flora.

## Introduction

Poultry production is an enormous industry in China, and more than 15.74 billion chickens were fed in 2021. Indeed, the laying hens are known for their long lifespan, and achieving a production cycle of 100 weeks with 500 eggs has been a productive goal. As intestinal injury in the chick phase can have irreversibly adverse effects on the growth and production performance in subsequent phases, the importance of maintaining gut health for layer chicks has garnered increasing attention. In practical production, the incidence of diarrhea and intestinal injury in chicken flocks reached 54.5 and 53.0% (Lobani et al., 2016; ter Veen et al., 2017), respectively, which reflects the serious intestinal health challenges in the poultry industry. In addition to the EU and US, China as the largest consumer of veterinary antibiotics has banned the addition of antibiotics in animal feed from 2020. Moreover, Ritter et al. (2019) reported that the mortality of broilers increased from 2.8 to 4.2% after antibiotics forbiddance, which makes a far more troubling set of intestinal health issues. Therefore, research focused on intestinal damage in a layer chick model, which has become even more prominent.

In fact, *Lactobacillus* was supposed to be a promising candidate for repairing intestinal injuries (Hou et al., 2018; Han et al., 2019). For the past few years, the positive benefits of *Lactobacillus plantarum* on intestinal damage have been verified in published research (Yang et al., 2020; Pupa et al., 2021). Otherwise, accelerating the succession of intestinal microbiota and achieving an equilibrium in microbiota much earlier in layers were supposed to be beneficial for the improvement of chick’s gut health (Dai et al., 2020). Given its promoting effects on intestinal flora succession, *L. plantarum* supplementation in the early phase of layer chicks probably exerts excellent function in alleviating intestinal damage. However, there are still some inconsistent reports that *L. plantarum* supplementation could not significantly improve the intestinal health status of animals (Lee et al., 2017). The foremost reason for these results may be that the survival of *L. plantarum* decreases drastically during the processes of *Lactobacillus* processing, storage, and digestion in the gastrointestinal tract of animals (Chan et al., 2010). In the previously published report, we established the microencapsulation technology of *L. plantarum* based on enzymatic hydrolysate of soybean protein isolate and modified phospholipid and found that *Lactobacillus plantarum* in microencapsulation (LPM) could pass through the gastric juice smoothly and sustain high livability when it arrived the intestine (Song et al., 2022). Therefore, compared with free *L. plantarum*, LPM supplementation could be supposed to be more accurate to monitor the alleviating effects of *L. plantarum* on intestinal damage. However, such research studies are limited, and more studies are needed to explore the effects of microencapsulated *L. plantarum* on intestinal injury in layer chicks.

The intestinal damage recovery could not be conducted without the proliferation and differentiation of intestinal epithelial stem cells (IESCs). However, reports concerning the effects of *Lactobacillus* on IESC proliferation and differentiation are very few, and more details are needed to be explored. Indeed, researchers have discovered two types of intestinal epithelial stem cells within the intestinal crypts. These include fast-cycling stem cells referred to as crypt base columnar (CBC) cells and quiescent stem cells known as +4 stem cells (Barker et al., 2007; Zhu et al., 2013). Among them, just above the crypt base, the quiescent ISECs are located at the so-called +4 position, and B-cell-specific Moloney murine leukemia virus insertion site 1 (Bmi-1) is a marker of these +4 stem cells (Smith et al., 2016). Yan et al. (2012) demonstrated that Bmi-1-marked quiescent ISECs could give rise to fast-cycling IESCs under stress conditions. By contrast, Lgr-5 (leucine-rich repeat-containing G protein-coupled receptor 5)-marked CBC cells were supposed to be active IESCs during homeostasis (Barker et al., 2007). Once Lgr-5+ daughter cells leave the crypt base, they will undergo a brief phase of proliferation within the transit amplifying (TA) zone while moving upward along the crypt–villus axis. Upon exiting the TA zone, these cells will become postmitotic and undergo terminal differentiation, and various functional cells are formed, such as absorptive enterocytes, goblet cells, enteroendocrine cells, and Paneth cells. These functional cells could replace the impaired intestinal epithelial cells and repair intestinal damage. Otherwise, *Lactobacillus* was reported to stimulate the proliferation and differentiation of intestinal epithelial stem cells *in vitro* (Hou et al., 2018). Based on the above, it can be speculated that the intestinal damage repairing of *L. plantarum* may be probably attributed to its acceleration of epithelial proliferation and differentiation. However, such research studies are a handful, and thus, more studies are needed.

In this study, the intestinal damage model was established through intraperitoneal lipopolysaccharide (LPS) injection, and then the effects of microencapsulated *L. plantarum* supplementation on the growth performance, intestinal development, intestinal morphology, viable count, microbiota, and epithelial cell proliferation and differentiation were investigated. Our findings may contribute to harnessing the potential benefits of microencapsulated *L. plantarum* in alleviating intestinal damage in layer chick production.

## Materials and methods

### Ethics statement

The experimental protocol was approved by the Animal Care and Use Committee of Henan University and Technology, and the methods were carried out in accordance with the relevant guidelines and regulations.

### Birds and experimental design

A total of 252 healthy Hy-Line Brown layer chicks (7-day-old with the same body weight) were randomly divided into six groups. Birds were injected with saline or LPS (300 μg/kg body weight) twice (7 and 10 days of age in the abdomen) except for the control, and the diets of birds subjected to LPS were supplemented with nothing, *L. plantarum*, LPM, and wall material of LPM (WM), respectively. LPM was prepared through *L. plantarum* M616 microencapsulation with WM, and the latter contained enzymatic hydrolysate of soybean protein isolate (4%; hydrolyzed by pepsin), modified phospholipid (10%), soybean oil (20%), sorbitol (60%), and glycerol (4%), according to the report (Song et al., 2022). The viable counts in LPM reached 10^9^ CFU/g. Hence, the specified supplemental levels of *L. plantarum*, LPM, and WM were 0.02 g (10^9^ CFU), 1.0 g, and 0.98 g, per kilogram feed, respectively. Each group had 7 replicates with 6 birds as a replicate, with *ad libitum* access to feed and water. Animal feeding was conducted according to the Feeding Management Manual of Hy-Line Brown layers. Experimental diets were formulated according to the Chinese Feeding Standard of Chicken (Wen et al., 2004) and NRC (1994). The formal feeding trial lasted for 8 days. Nutrient values of diets are measured and shown in Supplementary Table S1. The samples’ dry matter content was determined by drying at 105°C for 6 h, following the AOAC 2006 method (930.15). The Kjeldahl method (AOAC 2007; 976.05) was employed to analyze the crude protein. P content was analyzed using a spectrophotometer (UV-2700, Shimadzu, Japan), while Ca content was measured using a flame atomic absorption spectrophotometer (Zeenit700P, Analytik Jena, Germany).

### Growth performance and intestine morphology

Body weight was recorded at the beginning and end of the formal experiment. Feed intake was recorded every week. Average daily gain (ADG), average daily feed intake (ADFI), feed conversion ratio (FCR; feed: gain, both in grams), and mortality were calculated. At the end of the trial, 21 birds from each group were randomly selected (3 birds per replicate at average body weight) and weighed before slaughter. The weight and length of the duodenum, jejunum, and ileum were measured.

Segments (approximately 1.5 cm in length) in the middle portion of the duodenum, jejunum, and ileum (approximately 5 cm from Meckel’s diverticulum) were collected, washed with PBS, and fixed in 10% neutral-buffered formalin for histology analysis. The remainder of the ileum was opened longitudinally, and chyme samples were collected, immersed in liquid nitrogen, and then stored at −80°C for subsequent measurement. Intestinal epithelial mucosa tissue samples were collected, immediately immersed in liquid nitrogen, and then preserved at −80°C for the measurement of mRNA expressions of the investigated genes (Table 1). Moreover, the primers for these genes in layer chicks were designed and testified (Supplementary Figure S1). Duodenal, jejunal, and ileal samples were subjected to be washed, dehydrated, clarified, and embedded in paraffin. Serial sections were cut into 5 μm thickness, deparaffinized in xylene, rehydrated, stained with hematoxylin and eosin, fixed with neutral balsam, and observed by a light microscope (BX51, Olympus Co., Tokyo, Japan). The intestinal morphometry was evaluated by villus height (VH; from the tip of villus to the villus–crypt junction), crypt depth (CD; from the base up to the crypt–villus transition region), and the villus height to crypt depth ratio (VCR; Xie et al., 2014).

**Table 1:** Primer sequence of target and reference genes.

Gene^1^	Forward primer (5′-3′)	Reverse primer (3′-5′)	GenBank number	Product length (bp)
*IL-1β*	CCTCCAGCCAGAAAGTGAGG	TTGTAGCCCTTGATGCCCAG	XM_015297469.3	109
*IL-4*	GTGCCCACGCTGTGCTTAC	AGGAAACCTCTCCCTGGATGTC	AJ621249	82
*IL-6*	AGGGCCGTTCGCTATTTGAA	CAGAGGATTGTGCCCGAACT	NM_204628.2	72
*IL-8*	AGCGATTGAACTCCGATGCC	GCCATCTTTCAGAGTAGCTATGACT	NM_205018.2	136
*IL-10*	ACAAAGCCATGGGGGAGTTC	TAGCGGACCGAACGTTAAGC	NM_001004414.4	190
*IL-22*	ACGTCAACATCAGGGAGAACA	GGTACCTCTCCTTGGCCTCT	NM_001199614.1	99
*TNF-α* ^2^	GAGCGTTGACTTGGCTGTC	AAGCAACAACCAGCTATGCAC	NM_204267	
*IFN-γ* ^2^	AAAGCCGCACATCAAACACA	GCCATCAGGAAGGTTGTTTTTC	NM_205149.1	
*E-cadherin*	ACTGGTGACATTATTACCGTAGCA	TAGCCACTATGACATCCACTCTGT	NM_001001615	226
*Vil-1*	CTACCTCTGCGGGGATGAGC	CTGTTGGCGTAGCTGGTCTT	NM_001396564.1	141
*Lgr-5*	CCTTTATCAGCCCAGAAGTGA	TGGAACAAATGCTACGGATG	XM_425441.4	338
*ChA* ^3^	TGAATAAAGGGGACACTAAGG	AGCTCAGCCAGGGATG		337
*Lysozyme*	ATACAGCCTGGGAAACTGGGT	TACGGTTTGTAGCCTGGGTG	NM_205281.2	71
*Mucin-2*	TGTGGTCTGTGTGGCAACTT	GGCCTGAGCCTTGGTACATT	XM_046942297.1	357
*Bmi-1*	TTTCAAGATGGCCGCTTGGC	TGCACGTCTTGCAGAAGGAGT	NM_001007988.3	255
*PCNA*	GCAGATGTTCCTCTCGTTGTG	CGATGCTGCAATGCACTGAT	NM_204170.3	211
*Wnt-3*	TCCACAGCAAGGACAACGTA	ACGAGGGGTCTTTCACCCAT	XM_046904315.1	192
*Cyclin D1*	ACCCGACGAGTTACTGCAAA	TAGCGCACAGAGCCACAAAA	NM_001396513.1	173
*Olfm-4*	ACAACGACAGACGTGACTCC	GGAAAGGTGGTATCCGGCAA	NM_001040463.2	170
*c-Myc*	CCAGCAGCGACTCGGAAGAA	GGCTGGGTATTCCACCTTGG	NM_001030952.2	221
*Axin-2*	AGCCAAAGAAACTCCCGGTT	CAGTCAAACTCGTCGCTTGC	NM_204491.1	196
*Lrp-5*	GAGGGATGGGGCTGTGAAAAT	GTTGCCTCCCCAAATCCACT	XM_046918262.1	145
*Dll-1*	CGCCGCTATTGAGACACGAG	TCGTTGTTGTGGGCTGACTT	NM_001397475.1	75
*Notch-1*	GAGAAACGGCGACGGAACTT	CACACCTCCGTCCCATTGAG	NM_001397796.1	118
*Hes-1*	CACCGGAAGTCCTCCAAACC	GAGGTTCCTCAGGTGCTTCAC	NM_001005848	174
*AvBD-2*	CAAGGACTGCCTGCCACATA	ACCCTGGAGAAACCTGGAGT	NM_001201399.2	127
*AvBD-9*	ACACCGTCAGGCATCTTCAC	GGCAGGTCCCAATGTCAACT	NM_001001611.3	214
*AHR*	CGGAAACCTGTGCAGAAAATAGTAA	CATTCAAACGGTCCCTGTGC	NM_204118.3	88
*Claudin-1* ^2^	AAGTGCATGGAGGATGACCA	GCCACTCTGTTGCCATACCA	NM_001013611.2	
*Claudin-2*	TGAACCATTCGCAGTCCCTG	GGGAGGAGAGGTTACAGAGAT	NM_001277622.1	111
*Occludin* ^2^	TCATCGCCTCCATCGTCTAC	TCTTACTGCGCGTCTTCTGG	NM_205128.1	
*ZO-1^2^*	TATGAAGATCGTGCGCCTCC	GAGGTCTGCCATCGTAGCTC	XM_01527898.1	
*ZO-2*	TCAGCATTTCCAACCCTGCTA	TCTCAAGATGCTGAAGGACTGAA	XM_046934796.1	90
*ZO-3*	CCCCCAAGGACGGGTACA	TACACACTCGACACGAAGAT	XM_040692499.2	115
*β-actin* ^2^	GAGAAATTGTGCGTGACATCA	CCTGAACCTCTCATTGCCA	L08165	

^1^ZO-1, zonula occludens-1; ZO-2, zonula occludens-2; ZO-3, zonula occludens-3; PCNA, proliferating cell nuclear antigen; Lgr-5, leucine-rich-repeat-containing G-protein-coupled receptor 5; ChA, chromogranin A.^2^Sequences refer to Feng et al. (2021). ^3^Sequences refer to Zhao et al. (2022).

### Inflammatory factor measurement

At the end of the experiment, after fasting for 12 h, serum was obtained and stored at −20°C for the following analysis. After thawing at 4°C overnight, levels of interleukin (IL)-1β, IL-4, IL-6, and IL-10, tumor necrosis factor (TNF)-α, and interferon (IFN)-γ in serum were tested using ELISA kits for chicks (Shanghai Meilian Biological Technology Co., LTD., Shanghai, China), according to the manufacturer’s instructions. In addition, the gene mRNA relative expressions of inflammatory factors were measured, including *IL-1β*, *IL-4*, *IL-6*, *IL-8*, *IL-10*, *IL-22*, *TNF-α,* and *IFN-γ*.

### Short-chain fatty acid content and viable count analysis

Ileal digesta was used for short-chain fatty acid content and *L. plantarum* population analysis. Lactic acid content was measured according to the method by Borshchevskaya et al. (2016), and P-hydroxybiphenyl colorimetry was adopted in this research. Acetate, propionate, and butyrate concentrations were determined following the method described by Wielen et al. (2020), and the details were described in our former report (Cui et al., 2022). The amount of *L. plantarum* was determined by the dilution method of plate counting, and plate counts were performed by the spread plate method. The counting media were solidified using agar powder (18 g per liter). After thorough dispersion, the sample was diluted serially and plated in duplicate to obtain the viable count. *L. plantarum* was marked by fluorescein isothiocyanate (FITC), and the colonization of *L. plantarum* in the intestinal mucosal tissue was observed under a fluorescence microscope.

### Gut microbiota analysis

Microbial DNA was extracted from ileal content samples (approximately 0.3 g) taken from layer chicks using the E.Z.N.A Soil DNA Kit (Omega Bio-tek, Norcross, GA, USA). The integrity and quality of DNA samples were evaluated with 1% agarose gel electrophoresis and Nanodrop D-1000 spectrophotometer (Thermo Fisher Scientific, Waltham, MA, USA). Microbial 16S rDNA sequences spanning the hypervariable regions v3-v4 were amplified using forward primers: 338F (5’-ACTCCTACGGGA GGCAGCA-3′) and reverse primer: 806R (5’-GGACTACHVGGGTWTCTAAT-3′). The PCR reaction conditions were: 2 min of denaturation at 95°C; 25 cycles containing denaturation at 95°C for 30 s, annealing at 55°C for 30 s, and extension at 72°C for 30 s; and a final extension of 5 min at 72°C. Amplicons were extracted from 2% agarose gels and purified using the AxyPrep DNA Gel Extraction Kit (Axygen Biosciences, Union City, CA, USA) to remove superabundant primer dimers and dNTPs. Purified amplicons were qualified and sequenced using the MiSeq platform at Beijing Biomarker Biotechnology Co., Ltd. (Beijing, China). The raw reads were deposited to the NCBI Sequence Read Archive (SRA) database (Accession Number: PRJNA761001).

In microbial community analysis, raw paired-end sequences were carried out in Illumina HiSeq 2500. Following sequencing, raw data were converted to raw reads using base calling. After filtration (Trimmomatic v0.33) and screening (cutadapt 1.9.1), high-quality reads were collected. Then, they were pieced through overlap (FLASH v1.2.7) to obtain clean reads. Effective reads were obtained using the UCHIME (v4.2) after the identification and removal of chimera sequences. Then, these reads were clustered into operational taxonomic units (OTUs) with 97% sequence identity by Usearch (Edgar, 2013). Rarefaction curves and α-diversity analysis, including Shannon, Simpson, ACE, and Chao1 indices, were calculated using QIIME2. β-diversity was evaluated by computing the weighted UniFrac distance and visualized using principal coordinate analysis (PCoA).

### Goblet cell and PCNA^+^ cell percentage

The goblet cell percentage (GCP) was counted on 100 columnar cells of the villus. Otherwise, tissue sections were deparaffinized twice in xylene, rehydrated in a graded ethanol series, and rinsed in PBS (pH = 7.4) three times (5 min every time). The slides were incubated in a citrate acid buffer solution for 10 minutes using a steamer for heat-induced antigen retrieval. After samples were rinsed in PBS, 100 μL of H_2_O_2_ was supplemented to the zone marked by an immunohistochemical pen, and samples were incubated for 10 min at room temperature. Then, confining liquid, proliferating cell nuclear antigen (PCNA) antibody, antimouse IgG complex marked by biotin, and streptomyces antibiotin protein-peroxidase were added, and samples were incubated at room temperature. Diaminobenzidine was used as a color-substrate solution, while hematoxylin and resin were used to redye and seal. At 200 × magnification, the PCNA^+^ cell percentage was counted from the tip of the villus to the base of the crypt.

### Quantification of mRNA with real-time PCR

Total RNA was extracted with the TRIzol reagent (Tiangen Biotech CO., LTD, Beijing, China). The yield of RNA was determined using a NanoDrop 2000 spectrophotometer (Thermo Fisher Scientific, Waltham, MA, USA), and the integrity was evaluated using agarose-ethidium bromide electrophoresis. Quantification was carried out with a two-step reaction process: reverse transcription (RT) and PCR, according to the instructions of FastQuant RT Kit (KR106, Tiangen, Beijing, China). Each RT reaction consisted of 1 μg RNA. Real-time PCR was performed using a Light Cycler 480 Real-time PCR Instrument (Roche Diagnostics Ltd., Basel, Switzerland) with a 20 μL PCR reaction mixture that included 2 μL of cDNA, according to the instructions of the SuperReal PreMix Plus Kit (SYBR Green; KR106, Tiangen, Beijing, China). Reactions of real-time quantitative PCR were conducted in duplicate in the Bio-Rad C1000 thermal cycler (CFX-96 real-time PCR detection systems; Bio-Rad). The protocol used was as follows: 95°C for 15 min, 40 cycles of 95°C for 10 s, and 60°C for 30 s. The relative mRNA expression levels were normalized to avian β-actin with the 2^−ΔΔCt^ method (Livak and Schmittgen, 2001). Primer sequences are detailed in Table 2, and several of them refer to previous reports (Feng et al., 2021; Zhao et al., 2022).

**Table 2:** Effect of *Lactobacillus plantarum* supplementation on the growth performance of layer chicks^1^.

Items^2^	Control	Saline	LPS	LPS			SEM	*p*-value
				LP	WM	LPM		
Body weight, g								
7 d	81.75	82.08	87.25	85.83	84.83	83.83	1.14	0.73
14 d	157.75^ab^	153.75^ab^	150.75^b^	154.42^ab^	153.33^ab^	159.33^a^	0.87	0.041
d 7 to 14								
ADG, g	10.86	10.24	9.07	9.80	9.79	10.79	0.19	0.049
ADFI, g	23.79^a^	22.86^ab^	21.62^b^	22.76^ab^	22.14^b^	22.74^ab^	0.18	0.008
FCR	2.21	2.24	2.46	2.36	2.27	2.12	0.05	0.39

^1^Data are the mean of 7 replicates with 6 birds each.^2^LPS, Lipopolysaccharide; LP, *Lactobacillus plantarum*; LPM, *Lactobacillus plantarum* in microencapsulation; WM, wall material of LPM; ADG, average daily gain; ADFI, average daily feed intake; FCR, feed conversion ratio, feed/gain, g/g.^a–c^Values within a row with no common superscripts differ significantly (*p* < 0.05).

### Statistical analysis

SAS version 9.2 (SAS Institute, 2008) was used for statistical analyses. The experimental unit for growth performance analysis was the replicate, with each replicate in one cage. As for the other parameter measurements, the experimental unit for statistical analysis was the mean of three birds. The homogeneity of variances and normality of the data were first tested, and the normality was analyzed using the Shapiro–Wilk test. Then, a one-way ANOVA was conducted, and the means were compared using Tukey’s Multiple Range Test. Differences were considered statistically significant at *p* < 0.05, and the data were expressed as mean and pooled SEM.

## Results

### Growth performance

The effects of microencapsulated *L. plantarum* supplementation on the growth performance of layer chicks are presented in Table 2. At the beginning of the experiment, there was no significant difference in body weight among all treatments (*p* > 0.05). Otherwise, no significant differences were observed in ADG and FCR among all treatments (*p* > 0.05). Compared with the control, significantly lower values of ADFI were observed in the treatment with LPS alone and WM supplementation after LPS injection (WM/LPS; *p* < 0.05). The significantly higher values of body weight (14 d) occurred in LPM supplementation after LPS injection (LPM/LPS) treatment compared with LPS treatment (*p* < 0.05). Intestinal inflammation induced by LPS manifested as numerically lower values of body weight, ADG, and ADFI; meanwhile, *L. plantarum* supplementation could reverse this situation. Otherwise, compared with *L. plantarum* supplementation after LPS injection (LP/LPS) treatment, the numerically higher values of body weight (14 d) and ADG and the numerically lower values of FCR were simultaneously observed in LPM/LPS treatment, which indicated that LPM performed better than free *L. plantarum* in growth performance.

### Intestinal development

The development of the duodenum, jejunum, and ileum is shown in Table 3. No significant differences were observed in the indexes of the duodenum, jejunum, and ileum among all groups, as well as jejunum weight (*p* > 0.05). The significantly lower values of intestinal weight (the duodenum and ileum) and length (the duodenum, jejunum, and ileum) were observed in the treatments with LPS compared with the control (*p* < 0.05). In addition, compared with the control, significantly lower values were observed in WM/LPS treatment (*p* < 0.05). Meanwhile, compared with LPS treatment, intestinal weight (duodenum and ileum) and length (jejunum and ileum) significantly increased in LPM/LPS treatment (*p* < 0.05), as well as the significantly higher values of jejunal length in WM and free *L. plantarum* supplementation after LPS injection treatments (*p* < 0.05). The developments of intestinal weight and length were hindered by LPS injection; meanwhile, *L. plantarum* supplementation could alleviate these negative influences. Otherwise, compared with LP/LPS treatment, the numerically higher values of weight, index, and length of the duodenum, jejunum, and ileum were simultaneously observed in LPM/LPS treatment. Moreover, the higher ileal length occurred in LPM/LPS treatment compared with LP/LPS (*p* < 0.05; using Duncan’s multiple comparison). All of these findings indicated that LPM performed better than LP in the development of the small intestine.

**Table 3:** Effect of *Lactobacillus plantarum* supplementation on the development of small intestine in layer chicks (14 days of age)^1^.

Items^2^	Control	Saline	LPS	LPS			SEM	*p*-value
				LP	WM	LPM		
Duodenum								
Weight, g	2.37^a^	2.33^ab^	2.11^b^	2.24^ab^	2.19^ab^	2.35^a^	0.03	0.006
Index, %	1.51	1.52	1.40	1.45	1.43	1.47	0.02	0.25
Length, cm	15.33^a^	14.94^ab^	14.25^b^	14.80^ab^	14.44^ab^	15.01^ab^	0.11	0.024
Jejunum								
Weight, g	2.32	2.30	2.12	2.25	2.22	2.34	0.03	0.24
Index, %	1.47	1.50	1.41	1.45	1.45	1.47	0.02	0.76
Length, cm	27.47^a^	26.28^ab^	24.63^b^	27.02^a^	27.11^a^	27.38^a^	0.25	0.003
Ileum								
Weight, g	1.76^a^	1.72^a^	1.57^b^	1.65^ab^	1.62^ab^	1.75^a^	0.02	0.002
Index, %	1.12	1.12	1.04	1.07	1.05	1.10	0.01	0.13
Length, cm	27.18^a^	27.12^ab^	26.02^b^	26.30^ab^	26.05^b^	28.20^a^	0.22	0.021

^1^Data are the mean of 7 replicates with 3 birds each. ^2^LPS, Lipopolysaccharide; LP, *Lactobacillus plantarum*; WM, wall material of LPM; LPM, *Lactobacillus plantarum* in microencapsulation; BW, body weight; ADG, average daily gain; ADFI, average daily feed intake; F/G, feed/gain, g/g. ^a–c^Values within a row with no common superscripts differ significantly (*p* < 0.05).

### Intestinal morphology

**Figure 1:**
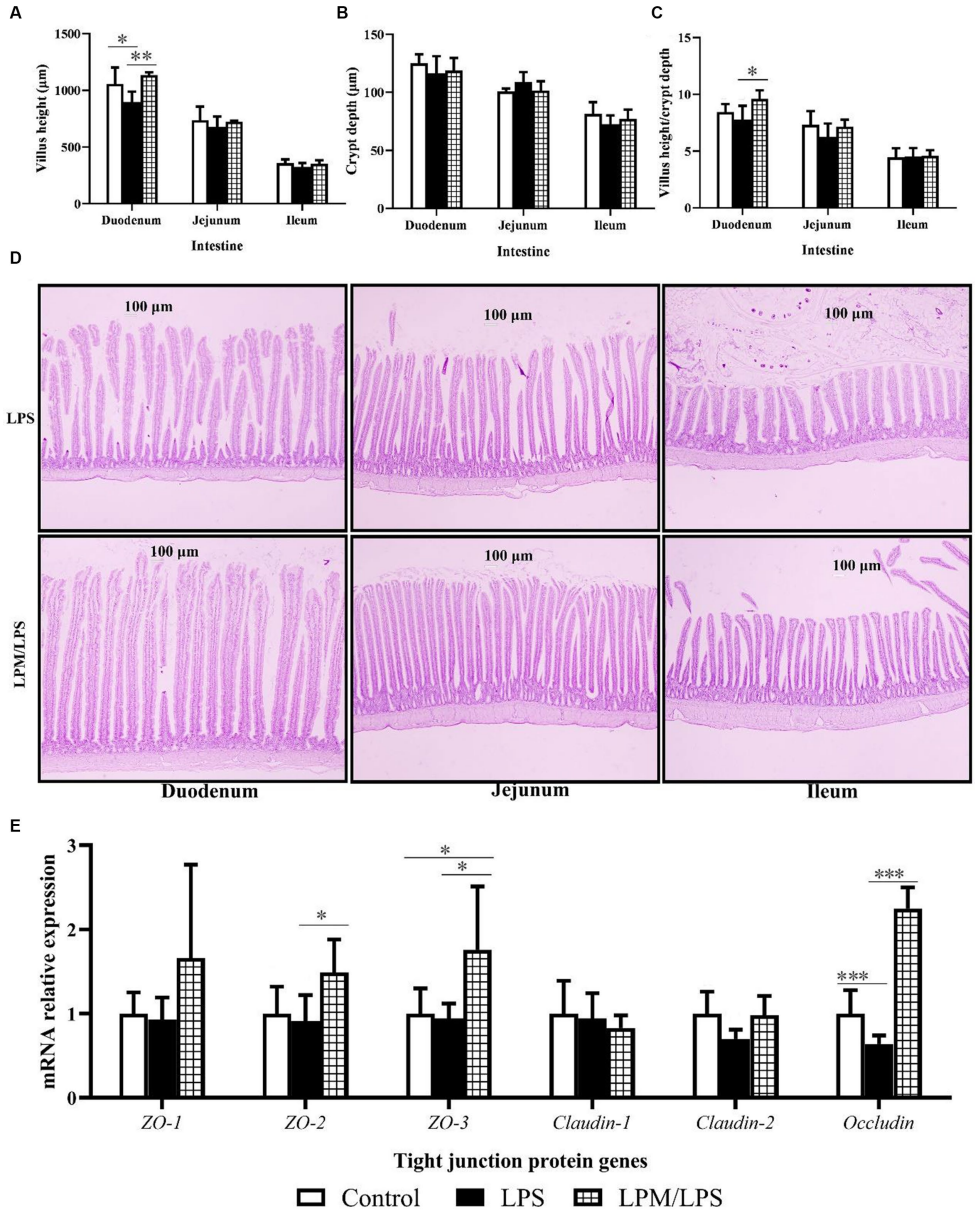
Effects of *Lactobacillus plantarum* in microencapsulation (LPM) supplementation on intestinal morphology (*n* = 7). **(A)** Villus height. **(B)** Crypt depth. **(C)** Villus height/crypt depth. **(D)** Intestinal morphology (100 ×). **(E)** The mRNA relative expressions of tight junction protein genes. Data are the mean of 7 replicates (2 chicks each replicate). LPS, Lipopolysaccharide; LPM/LPS, LPM supplementation after LPS injection. Differences were shown as ***(*p* < 0.001), **(*p* < 0.01), and *(*p* < 0.05), and the error bars mean standard deviation (SD).

The changes in intestinal morphology in response to the microencapsulated *L. plantarum* supplementation are detailed in Figures 1A–C. No significant changes were observed in crypt depth (duodenum, jejunum, and ileum) among all treatments (*p* > 0.05). Compared with the control, significantly lower values of the villus height in the duodenum were observed in treatment with LPS treatment (*p* < 0.05). Meanwhile, the significantly higher values of villus height and villus height/crypt depth in the duodenum were observed in LPM/LPS treatment compared with LPS treatment (*p* < 0.05). Typical intestinal longitudinal slices indicated better intestinal morphology was observed in LPM/LPS treatment compared with LPS treatment (Figure 1D). Otherwise, as shown in Figure 1E, no significant differences were observed in *zonula occludens-1* (*ZO-1*), *Claudin-1,* and *Claudin-2* mRNA relative expressions among all treatments (*p* > 0.05). Compared with the control, the significantly lower expressions of *Occludin* were observed in the treatment with LPS (*p* < 0.001), and the significantly higher expressions of *zonula occludens-3* (*ZO-3*) were observed in LPM/LPS treatment (*p* < 0.05). Moreover, compared with LPS treatment, the significantly higher expressions of zonula *occludens-2* (*ZO-2*), *zonula occludens-3* (*ZO-3*), and *Occludin* were observed in the treatment of LPM/LPS (*p* < 0.05).

### Inflammatory factor

**Figure 2:**
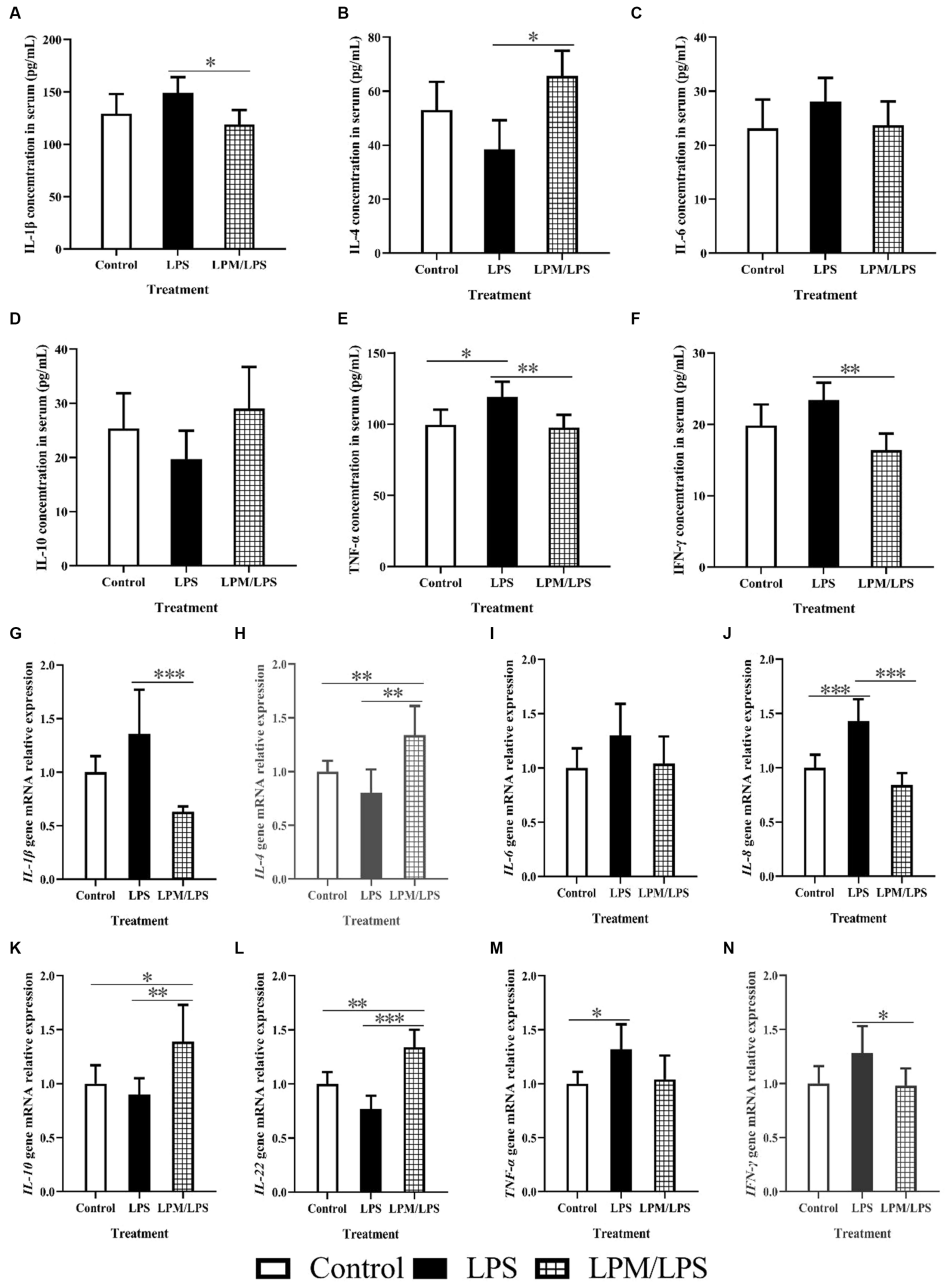
Effects of *Lactobacillus plantarum* in microencapsulation (LPM) on inflammatory factor (*n* = 7) **(A-N)**. LPS, Lipopolysaccharide; LPM/LPS, LPM supplementation after LPS injection. Differences were shown as ***(*p* < 0.001), **(*p* < 0.01), and *(*p* < 0.05), and the error bars mean standard deviation (SD).

The serum levels or gene mRNA relative expressions of main inflammatory factors, including IL-1β, IL-4, IL-6, IL-8, IL-10, IL-22, TNF-α, and IFN-γ, are detailed in Figures 2A–N. Among all the treatments, no significant differences were observed in IL-6 and IL-10 contents in serum and *IL-6* mRNA relative expression (*p* > 0.05). Compared with the control, LPS significantly increased TNF-α content in serum and the gene mRNA relative expressions of *IL-8* and *TNF-α* in ileal mucosal tissue (*p* < 0.05). Otherwise, compared with LPS treatment, LPM/LPS significantly decreased IL-1β, TNF-α, and IFN-γ contents in serum, and the gene mRNA relative expressions of *IL-1β*, *IL-8,* and *IFN-γ* in ileal mucosal tissue (*p* < 0.05) while increased *IL-4*, *IL-10*, and *IL-22* gene mRNA relative expressions in ileal mucosal tissue and IL-4 content in serum (*p* < 0.05).

### Intestinal pH, short chain fatty acid content, and *Lactobacillus plantarum* population

**Figure 3:**
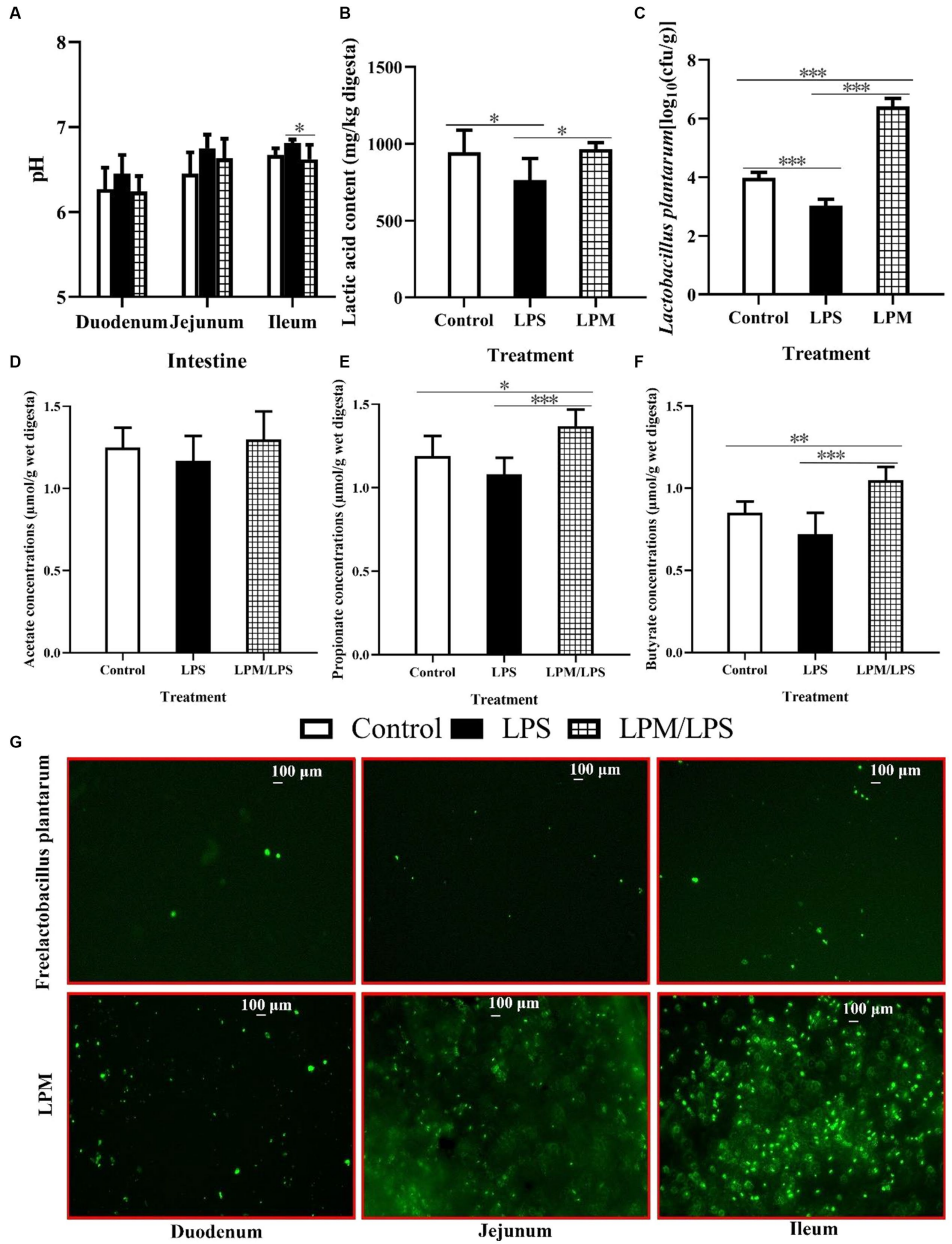
Effects of *Lactobacillus plantarum* in microencapsulation (LPM) supplementation on intestinal viable count. The values of pH **(A)** and ileal lactic acid content **(B)** and viable count of *L. plantarum* **(C)**, acetate **(D)**, propionate **(E)**, and butyrate **(F)** concentrations in digesta (*n* = 7). **(G)** *L. plantarum* colonization in the small intestinal mucosa of layer chicks in response to its supplemental form, which is marked by fluorescein isothiocyanate (FITC). LPS, Lipopolysaccharide; LPM/LPS, LPM supplementation after LPS injection. Differences were shown as *** (*p* < 0.001), **(*p* < 0.01), and *(*p* < 0.05), and the error bars mean standard deviation (SD).

The effects of LPM inclusion on intestinal pH, short chain fatty acid content, and *L. plantarum* populations in the ileum digesta are shown in Figures 3A–F. No significant differences were observed in the intestinal pH of the duodenum and jejunum among all treatments (*p* > 0.05), while the significantly lower ileal pH occurred in the treatment with LPM/LPS (*p* < 0.05) compared with LPS treatment. Otherwise, the significantly lower values of lactic acid content and *L. plantarum* number were observed in LPS treatment compared with the control (*p* < 0.05). Meanwhile, the significantly higher values of lactic acid, propionate, and butyrate levels and the *L. plantarum* population occurred in the treatment of LPM/LPS (*p* < 0.05) compared with the LPS treatment. Otherwise, fluorescence observation (marked by FITC) shown that LPM had visibly more *L. plantarum* in intestinal mucosa compared with that of free *L. plantarum* treatment (Figure 3G), indicating microencapsulation facilitated more viable *L. plantarum* arriving and colonizing in gut.

### Intestinal microbial diversity and community

**Figure 4:**
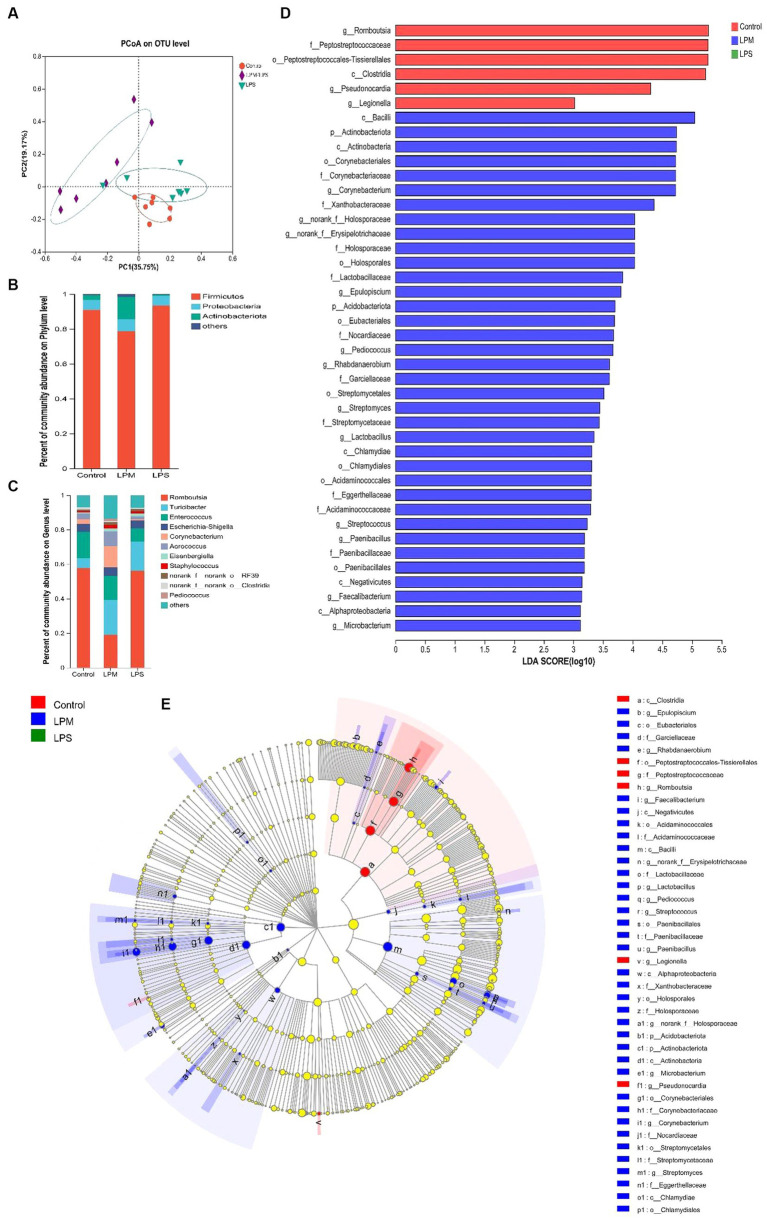
Effects of microencapsulated *Lactobacillus plantarum* supplementation on microbes in the ileal digesta of layer chicks (14 days of age; *n* = 7). LPS, Lipopolysaccharide; LPM, *Lactobacillus plantarum* in microencapsulation; LPM/LPS, LPM supplementation after LPS injection. **(A)** Principal coordinate analysis (PCoA) and composition of ileal microbiota [phylum **(B)** and **(C)** genus level]. **(D)** Histogram of the LDA scores computed for features differentially abundant among control (red bars), LPM/LPS (blue bars), and LPS (green bars) groups. Species with significant differences that have an LDA score of greater than 3.0 are presented. The length of the histogram represents the LDA score, which can be interpreted as the effect size of each differentially abundant feature. Data are the mean of 7 replicates. **(E)** Cladogram generated from Linear discriminant analysis (LDA) effect size (LEfSe) analysis, where red, green, and blue circles represent taxa of greater abundance in the control, LPS, and LPM/LPS groups, respectively. Yellow circles mean non-significant differences. The diameters of the circles are proportional to the abundance of the taxon.

After filtering, an average of 56,140 effective reads per sample were acquired. The sequencing depths were examined through the rarefaction curve for richness and numbers of shared OTU plotting. Moreover, the curves of most samples reached plateaus, indicating that the sampling depth was adequate. The effects of LPM supplementation on ileal microbial α-diversity of layer chicks are detailed in Table 4. No significant differences in the Shannon, Simpson, and ACE indices were observed among all treatments (*p* > 0.05). The significantly lower Chao1 index occurred in LPS treatment compared with the control (*p* < 0.05), while this situation ameliorated when LPM was supplemented. β-diversity analysis was conducted to compare the microbial profiles in ileal digesta among all treatments, as shown in Figure 4A. Principal coordinate analysis (PCoA) was first performed to show a holistic perception of the ileal microbiota. The results visually showed that these groups were mainly scattered into three clusters, which indicated that the microbiota compositions were quite dissimilar to each other.

**Table 4:** Effect of *Lactobacillus plantarum* supplementation on microbial α-diversity in ileum of layer chicks (14 days of age)^1^.

Items^2^	Treatments			SEM	*p*-value
	Control	LPS	LPM/LPS		
Shannon	1.69	1.63	2.30	0.15	0.124
Simpson	0.39	0.39	0.23	0.04	0.090
ACE	383.53	308.26	359.07	20.63	0.238
Chao1	372.82^a^	274.28^b^	347.85^ab^	20.63	0.049

^1^Data are the mean of 7 replicates (1 chick each replicate).^2^LPS, Lipopolysaccharide; LPM, *Lactobacillus plantarum* in microencapsulation; LPM/LPS, LPM supplementation after LPS injection.

To investigate the effect of LPM supplementation on the bacterial community members of ileal microbiota, the taxonomic compositions were explored at the phylum and genus levels. Three major phyla (Fimicutes, Proteobacteria, and Actinobacteria; relative abundance ˃ 1%) dominated the bacterial community (Figure 4B). Meanwhile, these phyla could be allocated into 10 major genera (*Romboutsia*, *Turicibacter*, *Enterococcus*, *Escherichia-Shigella*, *Corynebacterium*, *Aerococcus*, *Eisenbergiella*, *Staphylococcus*, *norank_f_norank_o_RF39*, and *norank_f_norank_o_Clostridia_UCG-014*; relative abundance ˃ 1%; Figure 4C). Compared with the LPS treatment, the significantly higher values of the Actinobacteria phylum and the lower value of the *Romboutsia* genus were observed in the LPM/LPS treatment (*p* < 0.05; Table 5).

**Table 5:** Effect of *Lactobacillus plantarum* supplementation on microbial relative abundance components in the ileum of layer chicks (14 days of age)^1^.

Items^2^	Treatments			SEM	*p*-value
	Control	LPS	LPM/LPS		
Phylum (%)					
Firmicutes	91.02	93.63	79.66	2.56	0.053
Proteobacteria	5.58	5.23	6.60	1.35	0.920
Actinobacteria	2.97^ab^	0.83^b^	12.31^a^	2.04	0.041
Genus (%)					
*Romboutsia*	57.68^a^	55.10^a^	18.98^b^	5.05	0.001
*Turicibacter*	5.71	16.21	20.43	3.72	0.262
*Enterococcus*	15.34	7.63	14.11	2.22	0.331
*Escherichia-Shigella*	4.66	4.78	4.73	1.23	0.999
*Corynebacterium*	2.55	0.61	11.73	2.03	0.048
*Aerococcus*	3.25	1.68	8.38	1.49	0.161
*Eisenbergiella*	0.78	1.81	1.92	0.60	0.715
*Staphylococcus*	0.79	1.69	2.06	0.42	0.475
*norank_f__norank_o__RF39*	0.82	1.45	1.70	0.34	0.567
*norank_f__norank_o__Clostridia_UCG-014*	1.17	0.93	0.74	0.21	0.735

^1^Data are the mean of 7 replicates (1 chick each replicate).^2^LPS, Lipopolysaccharide; LPM, *Lactobacillus plantarum* in microencapsulation; LPM/LPS, LPM supplementation after LPS injection.^a,b^Values within a row with no common superscripts differ significantly (*p* < 0.05).

Linear discriminant analysis (LDA) effect size (LEfSe) analysis was applied to identify the significantly differentially abundant OTUs for the entire ileal microbiota at the levels from phylum to genus (LDA > 3.0). As shown in Figures 4D,E, ileal discrepant microbiota in the control was mainly enriched with Clostridia class, Peptostreptococcales-Tissierellales order, and its downstream microbes (Peptostreptococcaceae and *Romboutsia*). The increased abundances of mainly discrepant microbes in the LPM/LPS group were as follows: phylum Actinobacteriota (Actinobacteria); class Negativicutes (Acidaminococcales, Acidaminococcaceae) and Chlamydiae (Chlamydiales); order Paenibacillales (Paenibacillaceae, *Paenibacillus*), Holosporales (Holosporaceae, *norank_f_Holosporaceae*), Corynebacteriales (Corynebacteriaceae, *Corynebacterium*), and Streptomycetales (Streptomycetaceae, *Streptomyces*); and family Lactobacillaceae (*Lactobacillus*).

### Intestinal epithelial cell proliferation and differentiation

**Figure 5:**
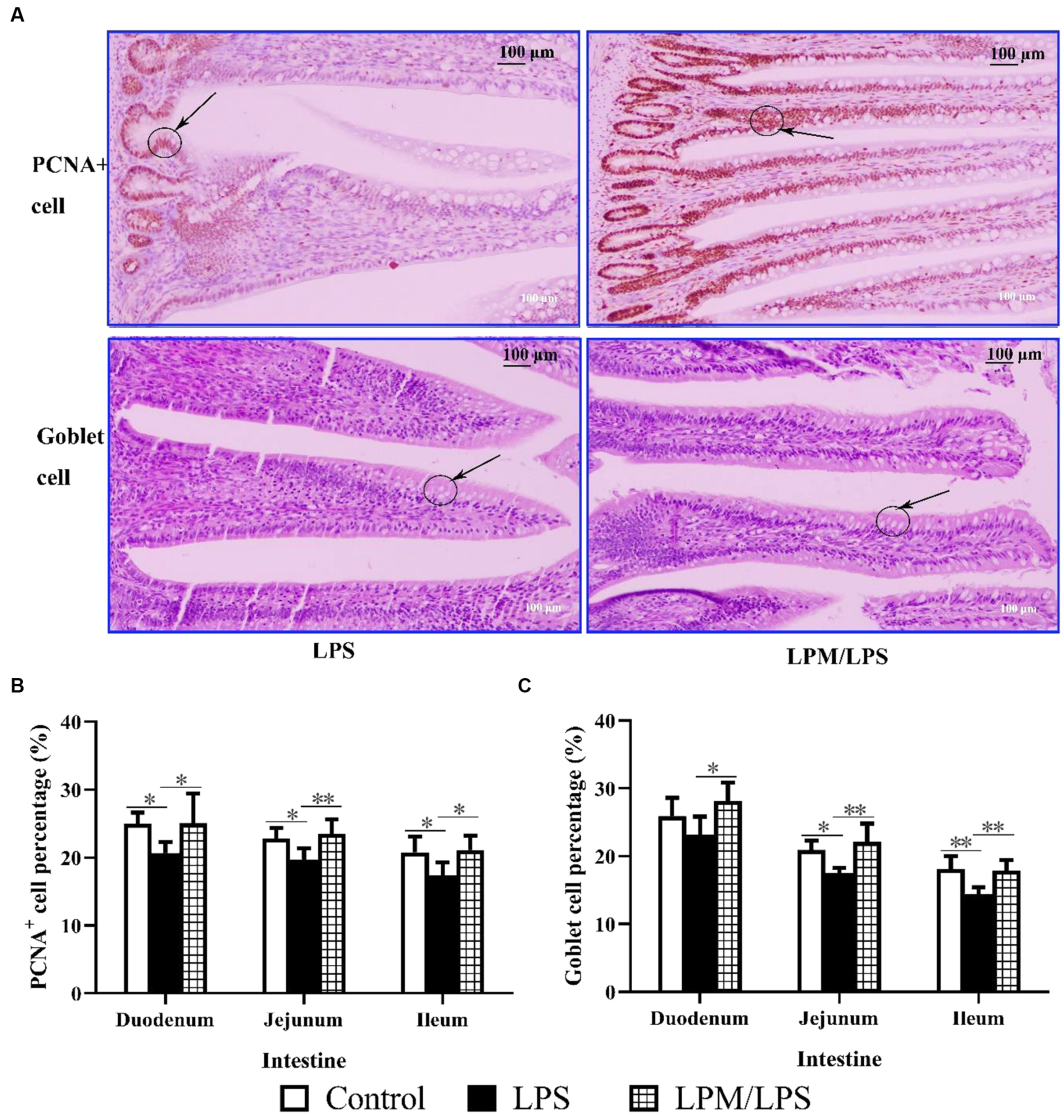
Goblet cell and PCNA+ cell percentage in intestinal epithelial tissue (*n* = 7). **(A)** The image (200 ×) of goblet cell and PCNA+ cell in the ileum of layer chicks in response to LPS and LPM supplementation (14 days of age). LPS, lipopolysaccharide; LPM, *Lactobacillus plantarum* in microencapsulation. Goblet cell **(B)** and PCNA+ cell **(C)** percentages in the epithelial tissue of the small intestine. Differences were shown as **(*p* < 0.01) and *(*p* < 0.05), and the error bars mean standard deviation (SD).

As shown in Figure 5A, PCNA+ cell and goblet cell percentages were visually higher in the LPM/LPS treatment than that of the LPS group, and these two cells reflect the proliferation and differentiation of intestinal epithelial stem cells. Compared with the LPS treatment, significantly higher PCNA+ cell and goblet cell percentages were observed in the duodenum, jejunum, and ileum of the LPM/LPS treatment (Figures 5B,C; *p* > 0.05). Obviously, the negative effects of LPS injection on intestinal epithelial stem cell proliferation and differentiation could be changed when microencapsulated *L. plantarum* was supplemented.

**Figure 6:**
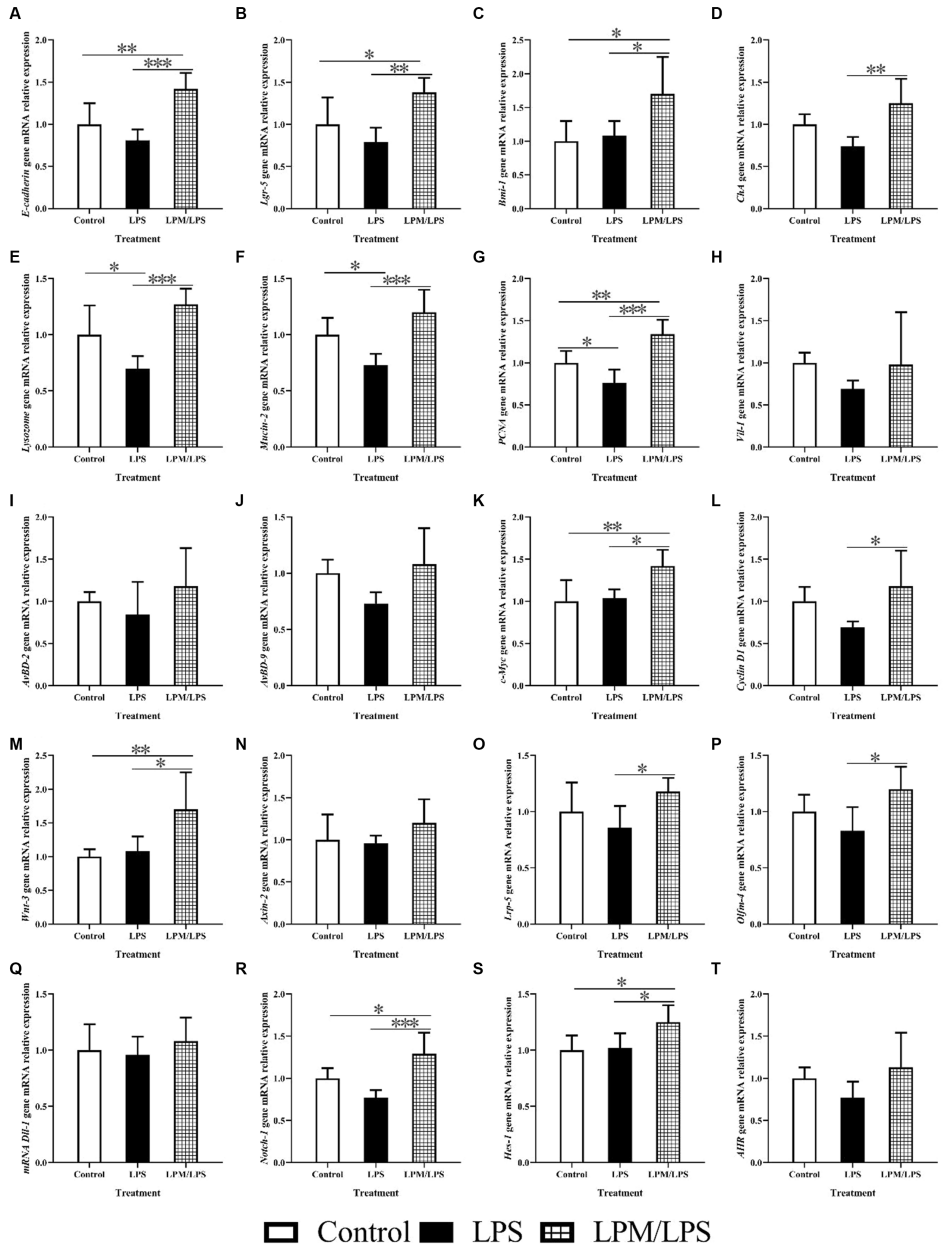
The mRNA relative expression of intestinal epithelial cell markers genes, intestinal epithelial cell proliferation and differentiation key genes in the ileum of layer chicks (14 days of age; *n* = 7) **(A–T)**. LPS, lipopolysaccharide; LPM, *Lactobacillus plantarum* in microencapsulation; LPM/LPS, LPM supplementation after LPS injection. *Lgr-5*, leucine-rich-repeat-containing G-protein-coupled receptor 5; *PCNA*, proliferating cell nuclear antigen. *ChA*, Chromogranin A. Differences were shown as ***(*p* < 0.001), **(*p* < 0.01), and *(*p* < 0.05), and the error bars mean standard deviation (SD).

The variations of intestinal epithelial cell marker gene mRNA relative expression in response to microencapsulated *L. plantarum* supplementation are detailed in Figures 6A–J. No significant differences were observed in the gene mRNA expressions of *Vil-1*, *AvBD-2,* and *AvBD-9* among all treatments (*p > 0.05*). Compared with the control, the significantly lower values of *Lysozome*, *Mucin-2,* and *PCNA* gene mRNA relative expressions were observed in LPS treatment. Meanwhile, the significantly higher values of *E-cadherin*, *Lgr-5*, *Bmi-1*, *ChA*, *Lysozome*, *Mucin-2,* and *PCNA* gene mRNA relative expressions occurred in the LPM/LPS treatment compared with the LPS treatment (*p* < 0.05).

In addition, the key gene mRNA relative expression during the process of intestinal epithelial stem cell proliferation and differentiation is shown in Figures 6K–T. No significant differences were observed in *Axin-2*, *Dll-1,* and *AHR* mRNA relative expressions among all treatments (*p* > 0.05). Compared with the LPS treatment, the mRNA relative expressions of *c-Myc*, *Cyclin D1*, wnt-3, *Lrp-5*, *Olfm-4*, *Notch-1,* and *Hes-1* increased in the LPM/LPS treatment (*p* < 0.05).

## Discussion

The positive effect of *L. plantarum* on intestinal health has been widely reported in various animals, such as pigs, broilers, and laying hens (Xu et al., 2020; Yang et al., 2020; Pupa et al., 2021). However, there are still some results that *L. plantarum* did not show obvious positive effectiveness on the gut health of animals (Lee et al., 2017), which may be due to the sharp decrease in the survival rate of *L. plantarum* during the process of reaching the bowel lumen (Chan et al., 2010). Based on our former findings that *L. plantarum* in the form of microencapsulation substantially enhanced its survival rate in artificial intestinal juice after flowing through gastric juice (Song et al., 2022), the effects of LPM supplementation on the growth performance and intestinal inflammatory damage repair of layer chicks were further investigated in this study. The intestinal damage model was induced successfully by intraperitoneal LPS injection, which could be evidenced by the significantly lower body weight, ADG, and ADFI during this experiment. Such findings may be due to the poor digestion and absorption function caused by intestinal damage. In fact, LPS injection caused intestinal damage had been reported in broilers before (Csernus et al., 2020). Based on the previous progress, the method of LPS injection in layer chicks was established in this research. In addition, compared with the free *L. plantarum* and WM, LPM supplementation exhibited a better improvement effect on the growth performance of layer chicks. These findings may be attributed to the speculation that microencapsulation could help *L. plantarum* smoothly pass through the adverse environment (such as gastric acid) and exert its benefits on intestinal digestion and absorption, which was consistent with the findings in our former report (Song et al., 2022). These results indicated that LPM effectively improved the growth performance of layer chicks that suffered intestinal damage.

The improvement of microencapsulated *L. plantarum* on growth performance to counteract LPS-induced decline could be attributed to the positive effect of LPM on intestinal development and intestinal morphology, which was further explored to illuminate the intestinal damage repair effect of microencapsulated *L. plantarum*. After birds received LPS injection, the development of their small intestine was suppressed, evidenced by the significant decrease in weight or length of different intestinal segments. Similar to the response of growth performance, LPM supplementation exhibited the better repair effect on the development of the small intestine in layer chicks compared with the free *L. plantarum* and WM. Taking the acceleration of *L. plantarum* on intestinal development (Wang et al., 2019) into consideration, these results may be attributed to more live *L. plantarum* arriving in the intestine brought by LPM. Such findings were consistent with the previous report that *Lactobacillus reuteri* in microencapsulation could enhance its survival in the intestines of broilers (Rodklongtan et al., 2014). Small intestine morphology is closely related to intestinal development. It is worth noting that LPS had adverse effects on small intestine morphology, appearing as significantly lower villus height, villus height/crypt depth, and tight junction protein expressions, including claudin-2 and occluding (using Ducan’s multiple-range test). In fact, tight junction proteins are mainly divided into three categories: occludin, claudin, and zonula occludens, and they constitute the epithelial barrier combined with the actin in the intestinal epithelial cell (Heinemann and Schuetz, 2019). The integrity of intestinal epithelial tissue can be reflected by tight junction proteins. In addition, compared with the control, LPS simultaneously increased proinflammatory factor expressions in the serum and ileum mucosa tissues, such as IL-1β, IL-6, IL-8, TNF-α, and INF-γ (using Ducan’s multiple-range test), which was consistent with the previous reports (Chan et al., 2017; Bang et al., 2022; Liu et al., 2022). Compared with the intestinal inflammation induced by LPS, microencapsulated *L. plantarum* significantly enhanced villus height, villus height/crypt depth, and tight junction protein expressions (ZO-2, ZO-3, and occludin) of layer chicks, accompanied by the increase in anti-inflammatory factors (IL-4, IL-10, and IL-22), and decreased proinflammatory factor (IL-1β, IL-8, TNF-α, and IFN-γ) expressions in the serum and ileum mucosa tissues. Such findings implied the outstanding intestinal inflammation repair function of LPM. All of these demonstrated that LPM could alleviate intestinal inflammatory damage of layer chicks induced by LPS, which could be probably attributed to the sufficient viable count of *L. plantarum* arriving in the gut.

Hence, the changes in intestinal microbiota were further investigated, as well as the viable counts of *L. plantarum*, in response to microencapsulated *L. plantarum* supplementation. The significantly higher viable microorganisms accompanied by higher lactic acid content and lower pH value were steadily observed in LPM treatment. The decrease in the intestinal pH could be attributed to the increase in intestinal lactic acid content. Lactic acid is the representative metabolite of *L. plantarum* (Passos et al., 1994), and thus the enhanced lactic acid content could be ascribed to the increase in viable *L. plantarum* colonization in the gut. Otherwise, significant increases were observed in propionate and butyrate, which was consistent with the previous report that *Lactobacillus plantarum* HNU082 increased propionate and butyrate concentrations in mice, indicating more viable *L. plantarum* colonizing in the gut (Wu et al., 2022). This speculation could be visibly verified by fluorescent observation based on FITC-marked *L. plantarum*. All of these demonstrated that LPM enhanced the viable count of *L. plantarum* in the gut.

As intestinal microbiota, in the α-diversity analysis, the Shannon and Simpson estimators were used to evaluate the microbiota diversity, while the ACE and Chao1 indices were adopted to reflect the microbiota richness (Ballou et al., 2016). In our findings, the Chao1 indicator displayed lower values in the LPS treatment group, suggesting a reduction in the microbial richness in the ileum of layer chicks when they were administered LPS injection. After LPM supplementation, the microbiota α-diversity increased in the ileum, which was evidenced by the numerical increase in the Shannon, ACE, and Chao1 indices, as well as a decrease in the Simpson estimator. The higher level of species diversity accompanied by a higher level of microbiota richness indicated a more stable microbiota community, which could prevent the colonization of pathogens and thus be beneficial to the gut health of the host bird (Han et al., 2018). Moreover, the lower microbiota α-diversity indicated poor productivity, which was consistent with the results that growth performance significantly decreased in LPS treatment. In addition, principal coordinate analysis (PCoA) was used to elucidate the β-diversity of ileal microbiota. The results showed significant clustering according to experimental groups, which demonstrated that ileal microbial community structure was significantly affected by LPS and LPM supplementation. Therefore, we speculated that the ileal microbiota of layer chicks could probably vary significantly in response to LPM inclusion, and variations in microbial composition and some specific taxons were further explored. Data showed that Actinobacteria at the phylum level increased in ileal microbial composition, while *Romboutsia* at the genus level decreased, with LPM supplementation after LPS injection. Given the benefits of Actinobacteria (Massot-Cladera et al., 2020) and the adverse effects of Romboutsia (Yang et al., 2021), these results indicated that LPM supplementation probably optimized ileal microbiota composition. In addition, LPM/LPS increased *Corynebacterium* at the genus level (using Ducan’s multiple-range test), which exhibited an inhibitory effect on inflammatory injury (Horn et al., 2022). The predominant microorganism species in the composition of ileal microbiota from the LPM/LPS treatment were consistent with the representative microbes obtained from LEfSe analysis of ileal microbiota in this group. In addition, Lactobacillaceae at the family level and *Lactobacillus* at the genus level stood out as the representative microbes in the LPM/LPS treatment. These findings were consistent with the former results that the higher viable counts of *L. plantarum* were observed in the LPM/LPS treatment. Therefore, it could be speculated that the alleviating effects of *L. plantarum* on intestinal damage induced by LPS could be attributed to the viable count enhancement and microbial composition optimization.

In fact, *Lactobacillus reuteri* D8 could stimulate the proliferation and differentiation of intestinal epithelial stem cells *in vitro* (Hou et al., 2018). Moreover, the increase in the intestinal villus height may be due to the proliferation and differentiation of intestinal epithelial stem cells and the migration of new cells along the villous-crypt axis (Parker et al., 2017). Therefore, the proliferation and differentiation of intestinal epithelial stem cells in response to LPM were further explored in this research. In fact, various intestinal epithelial functioning cells can conduct intestinal damage repair function, which derives from the proliferation and differentiation of intestinal epithelial stem cells. Hence, intestinal epithelial functioning cell marker genes were further investigated in this research, as well as the key genes involved in the process of intestinal epithelial stem cell proliferation and differentiation. In this research, *E-cadherin*, *Lgr-5*, *Bmi-1*, *ChA*, *Lysozome*, *Mucin-2*, *PCNA,* and *Vil-1* gene mRNA relative expressions were measured in this research, which were supposed to serve as the markers for the epithelium, fast-cycling stem cell, quiescent stem cell, endocrine cell, Paneth cell, goblet cell, proliferative cell, and absorptive cell (Feng et al., 2012; Jones et al., 2019; Xie et al., 2019). In this research, compared with LPS treatment, significantly higher values of *Lgr-5*, *Bmi-1,* and other epithelial functioning cell marker gene mRNA relative expressions simultaneously occurred in the LPM/LPS treatment, which was consistent with the previous report that quiescent intestinal epithelial stem cell could give rise to fast-cycling stem cell under stress condition (Yan et al., 2012). Meanwhile, the PCNA+ cell and goblet cell represent the proliferation and differentiation of intestinal epithelial stem cells, respectively, and the enhanced percentages of these two cells were consistent with the increase in the counts of intestinal epithelial functioning cells. These findings indicated that LPM alleviated intestinal damage in layer chicks probably by promoting the proliferation and differentiation of intestinal epithelial stem cells.

**Figure 7:**
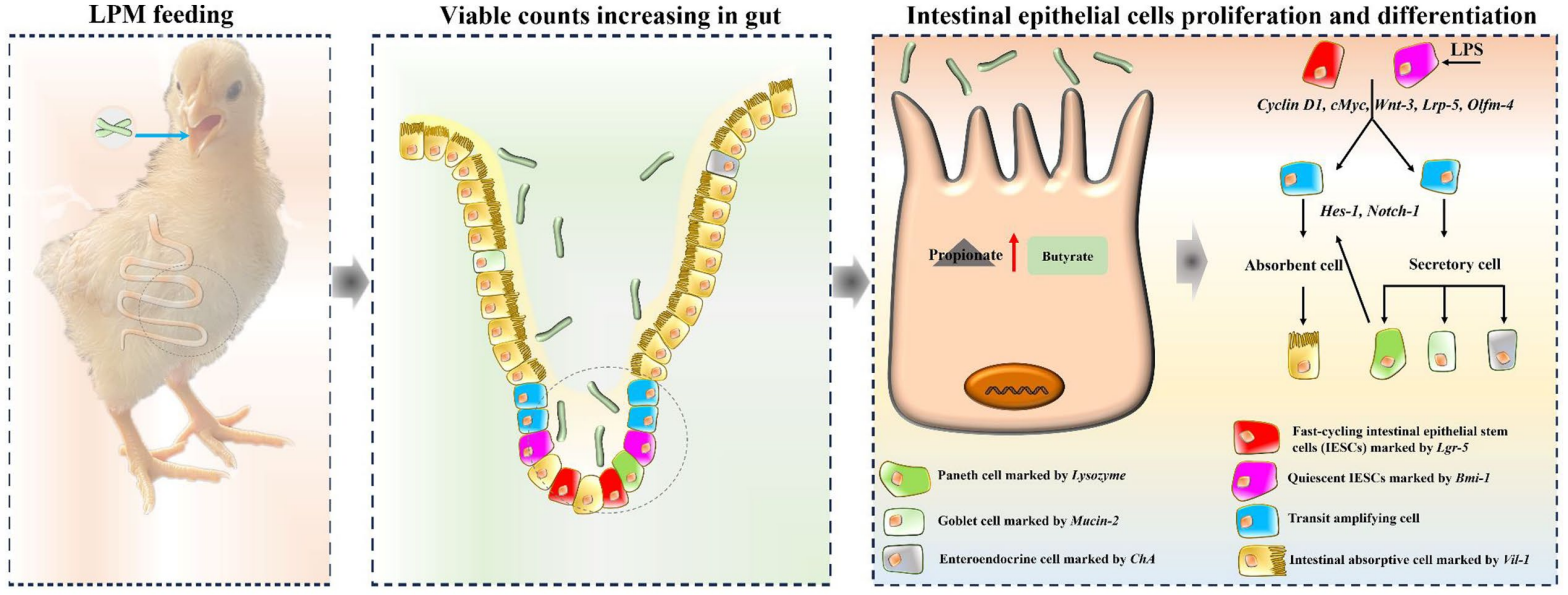
A schematic model displaying the potential mechanism of *L. plantarum* in microencapsulation promoting epithelial cell proliferation and differentiation in layer chicks.

These increased intestinal epithelial cells derived from epithelial stem cells, and thus, the key genes during the proliferation and differentiation process of stem cells were further explored. Variations caused by microencapsulated *L. plantarum* supplementation in the current research showed that *c-Myc*, *Cyclin D1*, *Wnt-3*, *Lrp-5,* and *Olfm-4* exerted significant effects during intestinal epithelial proliferation, while *Notch-1* and *Hes-1* played pivotal roles in intestinal epithelial differentiation. These findings were consistent with the former reports that *c-Myc*, *Cyclin D1*, *Wnt-3*, and *Lrp-5* promoted intestinal cell proliferation (Yamaguchi et al., 2004; Xie et al., 2019), while *Notch-1* and *Hes-1* stimulated epithelial cell differentiation in the gut (Xie et al., 2019). These findings could be attributed to the increased propionate and butyrate contents, which could provide fuel for intestinal cell proliferation and differentiation (Robles et al., 2013; Rodríguez-Enríquez et al., 2021). The potential mechanisms of LPM-promoting intestinal cell proliferation and differentiation in layer chicks are presented in Figure 7. Based on the above analyses, it could be supposed that microencapsulated *L. plantarum* could promote intestinal epithelial stem cell proliferation and differentiation, increase intestinal epithelial function cells, and alleviate intestinal inflammatory injury of layer chicks.

## Conclusion

In conclusion, microencapsulated *L. plantarum* could alleviate intestinal inflammatory damage in layer chicks induced through LPS administration, evidenced by the significantly decreased levels of pro-inflammatory factors, increased levels of anti-inflammatory factors, better growth performance, small intestine development, and intestinal morphology. Such findings could be due to the viable count enhancement of *L. plantarum* in the gut and intestinal microbiota composition optimization, which subsequently promoted the proliferation and differentiation of intestinal epithelial cells.

## Data availability statement

The datasets presented in this study can be found in online repositories. The names of the repository/repositories and accession number(s) can be found in the article/Supplementary material.

## Ethics statement

The animal study was approved by Animal Care and Use Committee of Henan University and Technology. The study was conducted in accordance with the local legislation and institutional requirements.

## Author contributions

YC: Formal analysis, Funding acquisition, Writing – review & editing, Writing – original draft. PH: Writing – review & editing, Project administration. HD: Supervision, Writing – review & editing. SS: Project administration, Writing – review & editing. LG: Formal analysis, Writing – review & editing. ZL: Project administration, Writing – review & editing. QL: Project administration, Writing – review & editing. JW: Supervision, Writing – review & editing. GQ: Supervision, Writing – review & editing. JG: Supervision, Writing – review & editing.
