# Supplementary material for: Role of microencapsulated Lactobacillus plantarum in alleviating intestinal inflammatory damage through promoting epithelial proliferation and differentiation in layer chicks
Source: Front Microbiol. 2023 Nov 20;14:1287899. doi: 10.3389/fmicb.2023.1287899 (PMC10694250; doi:10.3389/fmicb.2023.1287899)
Supplement: Supplementary file 1 [file Data_Sheet_1.pdf]

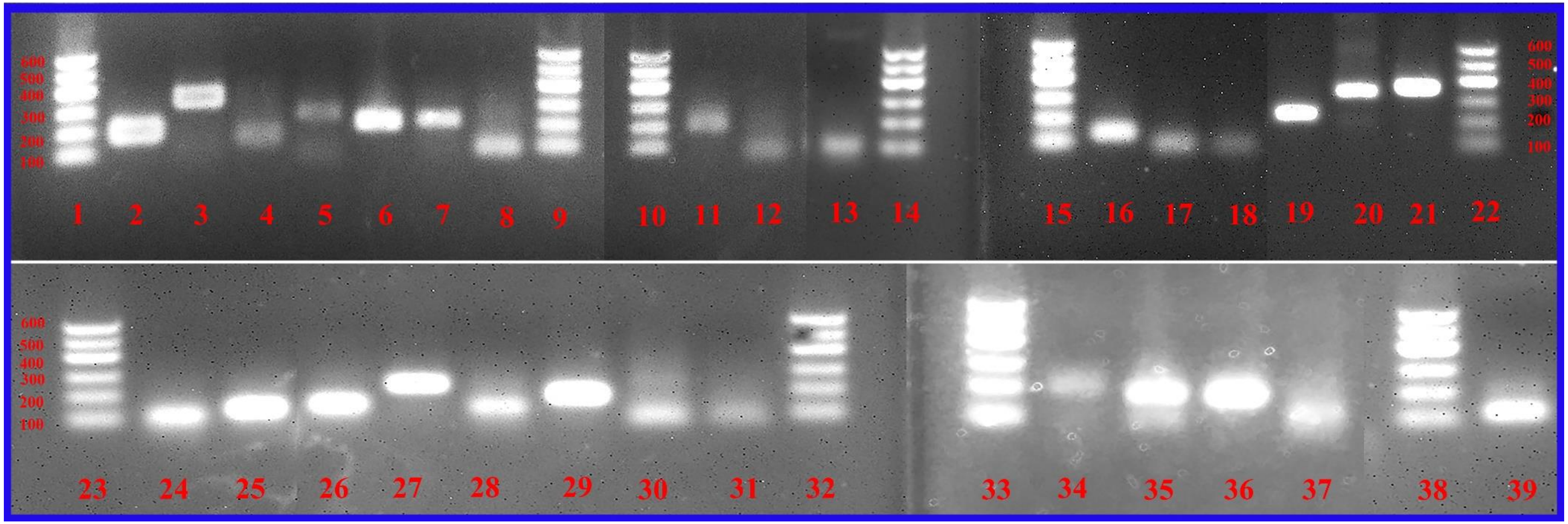

Figure S1. RT-PCR confirms key genes and their primers adopted in this research. A representative image of RT-PCR results from lane 1 to lane 39 are, DNA ladder (100, 200, 300, 400, 500 and 600 bp), *Axin-2* (196 bp), *Lgr-5* (338 bp), *Lrp-5* (145 bp), *Bmi-1* (255 bp), *PCNA* (211 bp), *c-Myc* (221 bp), *AHR* (88 bp), DNA ladder, DNA ladder, *Wnt-3* (192 bp), *ZO-2* (90 bp), *IL-22* (99 bp), DNA ladder, DNA ladder, *Vil-1* (141 bp), *IL-4* (82 bp), *IL-6* (72 bp), *E-cadherin* (226 bp), *ChA* (337 bp), *Mucin-2* (357 bp), DNA ladder, DNA ladder, *IL-1β* (109 bp), *IL-8* (136 bp), *AvBD-2* (127 bp), *AvBD-9* (214 bp), *Notch-1* (118 bp), *Hes-1* (174 bp), *Dll-1* (75 bp), *Lysozyme* (71 bp), DNA ladder, DNA ladder, *IL-10* (190 bp), *Cyclin D1* (173 bp), *Olfm-4* (170 bp), *Claudin-2* (111 bp), DNA ladder and *ZO-3* (115 bp), respectively.
