# Supplementary material for: Role of microencapsulated Lactobacillus plantarum in alleviating intestinal inflammatory damage through promoting epithelial proliferation and differentiation in layer chicks
Source: Front Microbiol. 2023 Nov 20;14:1287899. doi: 10.3389/fmicb.2023.1287899 (PMC10694250; doi:10.3389/fmicb.2023.1287899)
Supplement: Supplementary file 2 [file Data_Sheet_2.pdf]

Appendix A. Supplementary data

Table S1. Composition and nutrient content of basal diets of layer chicks (air-dry basis, %).

Items	Contents (%)
Ingredient	
Corn	63.07
Soybean meal	30.53
Corn gluten meal	2.00
Salt	0.30
Calcium phosphate	1.25
Limestone	1.30
DL-methionine	0.10
L-lysine	0.10
Choline chloride	0.15
Premix[1)]	1.00
Nutrient level[2)]	
AME (MJ/kg)	12.00
Crude protein	20.00 (20.28)
Calcium	0.95 (0.94)
Total phosphorus	0.65 (0.67)
Available phosphorus	0.41
Lysine	1.09
Methionine + cystine	0.77

[1] Premix supplied per kg of diet: vitamin A 12,500 IU, vitamin D_3 4,125 IU, vitamin E 15 IU, vitamin K_3 2 mg, thiamine 1 mg, riboflavin 8.5 mg, pyridoxine 8 mg, vitamin B_{12} 5 mg, biotin 2 mg, folic acid 5 mg, Ca-pantothenate 50 mg, niacin 32.5 mg, Cu 11 mg， Zn 65 mg, Fe 60 mg, Mn 51 mg, Se 0.16 mg, I 0.51 mg.

[2] The values in parentheses indicate the analyzed values. Others are calculated values.
